# Mediterranean Diet and Soy Isoflavones for Integrated Management of the Menopausal Metabolic Syndrome

**DOI:** 10.3390/nu14081550

**Published:** 2022-04-08

**Authors:** Herbert Ryan Marini

**Affiliations:** Department of Clinical and Experimental Medicine, University of Messina, 98125 Messina, Italy; hrmarini@unime.it; Tel.: +39-(090)-221-3328; Fax: +39-(090)-221-7001

The transition from premenopause to postmenopause is associated with the development of multiple elements of Metabolic Syndrome (MetS) [1,2,3]. The typical features of the syndrome [4,5] could be related to a progressive estrogen deficiency resulting in metabolic consequences of central fat redistribution associated with progressive testosterone predominance [2,3]. These changes lie with a dramatic increase in the risk of cardiovascular disease (CVD), which is the leading cause of death in women 65 years and older [3]. Prevention and treatment of MetS in menopause should focus on lifestyle modifications (i.e., dietary style and physical activity) [6].

Isoflavones and related compounds act through the estrogen receptors (ERs) and are often used in postmenopausal women [7]. Our research group previously showed that genistein, which is the most abundant and active isoflavone in soy, acting as a natural selective ER-β modulator [8], positively regulates bone metabolism [9,10], improves endothelium-dependent dilation [11], and reduces vasomotor symptoms [12] and some cardiovascular risk markers [13,14], without harmful side effects on thyroid [15] and reproductive tissues, including breast tissue [16]. Additionally, one year of treatment with pure genistein (54 mg/day) ameliorated cardiac and endothelial functioning [17,18], as well as improved surrogate endpoints associated with risk for diabetes and CVD in postmenopausal women with MetS [19].

The relationship between soy products, isoflavones, diet, and CVD has become a controversial topic.

Among plant-based diets, current evidence suggests that the Mediterranean and vegetarian diets are associated with numerous health benefits, including a lower risk of CVD [20]. These positive effects may be explained by their high content of dietary fiber, complex carbohydrates, vitamins, minerals, polyunsaturated fatty acids, and phytochemicals [20]. Specifically, a recent work by Dinu and colleagues of “The Working Group “Young Members” of the Italian Society of Human Nutrition (SINU)” suggested that the “Mediterranean diet had the strongest and most consistent evidence of a beneficial effect on both anthropometric parameters and cardiometabolic risk factors” [21].

So far, a very recent observational research published in Circulation by Le Ma and coworkers [22] indicated that “higher intake of isoflavones and tofu was associated with a moderately lower risk of developing Coronary Heart Disease, and in women the favorable association of tofu were more pronounced in young women or postmenopausal women without hormone use”; consequently, it appears that an adequate intake of isoflavones and/or soy products such as tofu can be integrated into healthy plant-based diets, adding nutritional support in the prevention of CVD [23]. However, as remarked by Carnethon and Khan in their editorial on time [24], “there are critical limitations of observational studies of diet that warrant caution about making causal statements”.

Undoubtedly, the question is interesting, opening new intriguing fields studying the effects of the combination “Mediterranean diet” and soy isoflavones intake, with the ambitious target to better define in post-menopausal women [25] the relationship between diet, bioactive foods, and nutraceuticals then counteracting, through lifestyle modifications, the constellation of risk factors that characterize MetS.
